# Strategies to Improve Drug Delivery Across the Blood–Brain Barrier for Glioblastoma

**DOI:** 10.1007/s11910-024-01338-x

**Published:** 2024-04-05

**Authors:** Kazim H. Narsinh, Edgar Perez, Alexander F. Haddad, Jacob S. Young, Luis Savastano, Javier E. Villanueva-Meyer, Ethan Winkler, John de Groot

**Affiliations:** 1 Department of Neurologic Surgery, University of California, San Francisco, CA USA; 2 Department of Radiology & Biomedical Imaging, University of California, San Francisco, CA USA

**Keywords:** Glioblastoma, GBM, Drug delivery, Convection-enhanced delivery, Laser interstitial thermal therapy, Focused ultrasound, Clinical trials

## Abstract

**Purpose of Review:**

Glioblastoma remains resistant to most conventional treatments. Despite scientific advances in the past three decades, there has been a dearth of effective new treatments. New approaches to drug delivery and clinical trial design are needed.

**Recent Findings:**

We discuss how the blood–brain barrier and tumor microenvironment pose challenges for development of effective therapies for glioblastoma. Next, we discuss treatments in development that aim to overcome these barriers, including novel drug designs such as nanoparticles and antibody–drug conjugates, novel methods of drug delivery, including convection-enhanced and intra-arterial delivery, and novel methods to enhance drug penetration, such as blood–brain barrier disruption by focused ultrasound and laser interstitial thermal therapy. Lastly, we address future opportunities, positing combination therapy as the best strategy for effective treatment, neoadjuvant and window-of-opportunity approaches to simultaneously enhance therapeutic effectiveness with interrogation of on-treatment biologic endpoints, and adaptive platform and basket trials as imperative for future trial design.

**Summary:**

New approaches to GBM treatment should account for the blood-brain barrier and immunosuppression by improving drug delivery, combining treatments, and integrating novel clinical trial designs.

## Introduction

Glioblastoma (GBM) is the most common malignant primary brain tumor in adults, accounting for approximately 46% of all primary central nervous system (CNS) malignancies, with near universal mortality. The prognosis for GBM remains poor with a calculated 5-year overall relative survival of only 6.8% [1]. In the last 25 years, only two drugs, temozolomide (TMZ) and bevacizumab (BEV), have been approved by the FDA for the treatment of high-grade gliomas, and BEV has subsequently been shown to not actually improve overall survival (OS) in newly diagnosed GBM [2]. The treatment arsenal for GBM includes surgery, radiation therapy, TMZ, tumor-treating fields, and a handful of other chemotherapies such as lomustine (CCNU) and carmustine (BCNU), but invariably these agents ultimately fail to provide long-term tumor control. In this review, we will describe the current treatment strategies for GBM, discuss challenges to drug delivery, highlight new approaches to therapeutic delivery across the blood–brain barrier (BBB), and elaborate on future directions for clinical trial design.

## Current Treatment

The current standard of care treatment protocol for newly diagnosed GBM is maximal safe surgical resection followed by fractionated radiotherapy to 60 Gy in 30 fractions with concurrent and adjuvant TMZ chemotherapy [3, 4]. Extent of resection (EOR) is a strong predictor of overall survival (OS) with greater than 98% EOR improving OS significantly to 13 vs. 8.8 months [5, 6]. In their landmark 2005 paper, Stupp et al. demonstrated that the addition of TMZ chemotherapy to radiation led to a 2-month increase in median OS and a doubling of 2-year OS rate [4]. This remains one of the most significant improvements in GBM survival achieved by a chemotherapeutic agent. BEV is an anti-vascular endothelial growth factor (VEGF) antibody approved by the FDA for use in recurrent disease and is primarily used to treat symptomatic edema and radiation necrosis. While phase 2 trials using BEV in recurrent malignant glioma showed a high radiologic response rate of up to 63%, this was due to VEGF sequestration leading to a reduction in vascular permeability and contrast-enhancement, subsequently termed a “pseudoresponse.” These encouraging results did not translate into a significant improvement in OS in recurrent or newly diagnosed glioblastoma trials [7–9]. Tumor-treating fields, an adjunct treatment that delivers electromagnetic pulses via a device worn on the head and that has been shown to interfere with mitotic bundling in vitro, have demonstrated a survival benefit in a randomized trial [10], although a systematic review of patients treated with maximal standard treatment that includes tumor-treating fields has only shown an extension of survival to a maximum of 20.7 months [3]. The remainder of the FDA-approved chemotherapies, CCNU and IV BCNU, have shown no benefit compared to radiation therapy alone [11, 12]. BCNU wafer implants (Gliadel) placed in the surgical cavity have been specifically criticized for their high complication rates, high expense, and limited therapeutic efficacy. Despite impressive scientific advancements in the past three decades, there remains a dearth of effective new drug treatments for GBM.

## The Biology of Glioblastoma

In this section, we discuss how GBM’s characteristic intra-tumoral heterogeneity, as well as the local and systemic immunosuppressive environment that it induces, has limited the development of effective therapies.

### Tumor Heterogeneity

The molecular landscape of GBM is highly heterogeneous. While The Cancer Genome Atlas (TCGA) initially identified four molecular subgroups of GBM (classical, neural, pro-neural, and mesenchymal) based on dominant genes expressed using transcriptional profiling data of bulk tumor specimens [13], at single-cell RNA-sequencing resolution, a single tumor actually consists of a heterogeneous mixture of cells representing all of the different GBM subgroups [14]. Moreover, these subgroups are dynamic and vary spatially and temporally within the same tumor [15]. Functional heterogeneity in GBM is further enhanced by epigenetic mechanisms [16]. Intra-tumoral heterogeneity through space and time, influenced by epigenetic changes, strongly contributes to therapy failure. Even when a promising target is identified, variable expression of the protein target makes treatments less efficacious than anticipated. Sub-clonal populations of cells with selectable traits constantly arise in response to therapy, providing lineages of therapy-resistant tumor cells. For instance, GBM acquires resistance to TMZ through alterations in their expression of DNA alkylating proteins, DNA repair enzymes, as well as cell signaling pathways [17].

### Systemic and Local Immunosuppression

A growing literature over the past decade has overturned preceding canon of the brain as an immune-privileged space, hinting at a role for immunotherapies in the treatment of neoplasms affecting the CNS [18]. Unfortunately, the treatment of GBM with single-agent immune checkpoint inhibitors has not resulted in significant benefits for patients to date [19]. Indeed, GBM is a uniquely immunosuppressive disease that leads to both local and systemic immune suppression in patients. Locally, GBM cells downregulate expression of MHC class I and MHC class II proteins [20, 21], limiting neoantigen presentation to the immune system. GBM also has fewer neoantigens for the immune system to identify and target compared to other solid malignancies given its low tumor mutation burden, and tumor mutational burden has been shown to correlate with response to immunotherapies in other cancer types [22–24]. Additionally, GBM maintains an immunosuppressive local microenvironment through a variety of signaling pathways, including the release of immunosuppressive cytokines (e.g., IL-6), the expression of immune checkpoint molecules (e.g., PD-L1), TGF-B signaling, and the expression of STAT3 [25–30]. These pathways can contribute to T cell tolerance, lead to the formation of regulatory T cells that are implicated in inhibiting T cell proliferation, block anti-tumor immune responses, and attenuate cytotoxic T cell activity [25–30]. In addition, GBM has relatively few tumor infiltrating T cells, and those that do infiltrate the tumor are exhausted and dysfunctional with a limited ability to mount an effective anti-tumor immune response [31, 32]. The myeloid compartment of GBM tumors, which represents the majority of the infiltrating immune cells, significantly contributes to the immunosuppressive local tumor environment through tumor-associated macrophages (TAMs) and myeloid-derived suppressor cells (MDSCs), which inhibit T cell activation and proliferation [33]. Unsurprisingly, intratumoral infiltration of MDSCs in patients with GBM has been correlated with patient survival, highlighting their potential as an immunotherapeutic target [34].

Though GBM is rarely metastatic beyond the CNS, it does elicit systemic immunosuppression that extends beyond the brain. The systemic effects of GBM include decreased T cell counts and functionality, small secondary lymphoid organs, and lower class II MHC expression levels systemically [31, 35]. CD4 T cell counts in pre-resection, pre-chemotherapy GBM patients have been found to approach levels comparable to those seen in AIDS patients [31, 35, 36]. While many of these patients had been treated with preoperative steroids, the extent of the drop in CD4 counts cannot solely be explained by steroids, and such drops have not been seen in patients receiving identical steroid regimens prior to surgery for other conditions. In mouse models of GBM and other intracranial tumors, missing T cells have been shown to be sequestered in the bone marrow secondary to the loss of S1P1 on the T cell surface [31]. This relative lymphopenia and immunosuppressive tumor microenvironment have led to the labeling of GBM as an immunologically “cold” tumor, making it difficult to target with immunotherapies.

In the past two decades, immunotherapy has shown tremendous promise in cancer treatment, especially in immunogenic cancers such as melanoma. However, immunotherapies often rely on targeting specific antigens and magnifying the immune system’s natural role in tumor surveillance and regulation. As such, they depend on a relatively homogenous tumor cell population and a competent immune system in order to be effective—two characteristics that are absent in patients with GBM. Several prominent clinical trials of immunotherapies in GBM have failed to demonstrate therapeutic benefit [19, 30, 37, 38]. These trials have primarily focused on systemically administered single-agent check point inhibition, which has a number of limitations including questionable penetrance of the blood–brain barrier and a failure to address the multifaceted immunosuppression present in GBM patients [19].

Additional immunotherapies under development include oncolytic viruses, cancer antigen vaccines, and CAR T cells. Oncolytic viral therapies utilized for the treatment of GBM are frequently replication competent viruses that lyse and kill tumor cells as they replicate, leading to the release of tumor associated antigens and localized inflammation such as adenovirus, poliovirus, herpes simplex virus, and reovirus [39]. Replicating retrovirus (RRV) has also been utilized to treat GBM and is unique in its ability to spread throughout a tumor without causing cell death, expanding potential therapeutic options. Clinical trials evaluating viral treatments for GBM have had limited success thus far. Toca 511 (vocimagene amiretrorepvec), a RRV which delivered a yeast cytosine deaminase suicide gene to GBM cells, has failed to demonstrate an overall survival benefit in a Phase III clinical trial, although the platform was safe [39]. A recent Phase II trial using repeated injections of G47∆, a triple-mutated, third-generation oncolytic herpes simplex virus, demonstrated an overall survival of 84% at 1 year after viral injections (in patients with an expected 1-year survival of 15%). Results from the trial also demonstrated increased infiltration of CD4 and CD8 T cells, hinting at the importance of a T cell-mediated anti-tumor immune response in their treatment effect, which is consistent with preclinical findings in multiple viral treatments [40].

CAR-T cells have had incredible benefits in hematologic malignancies but limited responses in solid tumors to date, including in GBM. CAR T cells targeting IL-13Rα2 and EGFRvIII have had limited responses in GBM due to the heterogenous nature of GBM and multiple immunosuppressive mechanisms present in the tumor microenvironment [30]. Vaccines for the treatment of GBM using tumor lysates have had limited success [30]. A recent Phase III study of autologous tumor lysate-loaded dendritic cell vaccination (DCVax-L) in recurrent and newly diagnosed GBM reported a survival benefit, although the study has been criticized for using historical data as for external control comparator [41].

## The Biology of the Blood–Brain Barrier

While therapy failure can in part be explained by the complex intra-tumoral heterogeneity and immunosuppressed state inherently associated with GBM, the BBB also mounts a significant challenge. The BBB consists of specialized, non-fenestrated endothelial cells in contact with pericytes and astrocytes, linked together by adherens junctions (e.g., vascular endothelial cadherin and platelet endothelial cell adhesion molecule-1), gap junctions (e.g., connexin-37), scaffolding proteins (e.g., zona occludens-1), and tight junctions (e.g., occludin and claudin-5). The BBB not only serves to isolate the brain from pathogens and toxins but also limits drug penetration to lipophilic, low molecular weight molecules (less than 400–500 Da). This means that approximately 98% of small molecules and nearly all large therapeutic molecules, such as monoclonal antibodies, antisense oligonucleotides, or viral vectors, cannot pass through this barrier. For example, while the standard-of-care TMZ falls within the parameters of permeability as a 194 Da lipophilic molecule, the highest tumor-to-blood concentration ratio achieved is < 20% [4, 42, 43]. Even chemicals that are penetrable can have limited concentrations because efflux pumps along the membrane such as p-glycoprotein (P-gp), breast cancer resistance protein (BCRP), and others, pump xenobiotics back into the systemic circulation.

GBM is radiologically characterized by areas of contrast enhancement due to gadolinium penetration across the BBB, leading to the belief that drugs also penetrate the contrast-enhancing regions of the tumor. Some studies have demonstrated that drug concentrations are higher in areas of contrast enhancement compared to non-enhancing areas [44, 45]. However, there are several challenges to this reasoning. First, gadolinium chelates are chemically dissimilar to most therapeutic agents. Second, these areas of the tumor are highly vascular, and drug levels may reflect drug accumulation within the vascular compartment. Third, although drug may penetrate these areas, it is possible that efflux pumps may re-distribute drug back into the systemic circulation. Finally, GBM cells invade areas of the brain with an intact BBB; in fact, much of the infiltrative component of the tumor is protected by an intact BBB [46]. This is in line with radiologic imaging of GBM, where infiltrative disease can appear as non-enhancing T2-weighted hyperintensity, and tumor cells can even extend into tissue with normal-appearing T2 signal intensity [47, 48]. This heterogeneity in the permeability of the BBB will critically impact pharmacodynamics, vary the distribution of treatment drugs, and ultimately limit their efficacy. For example, in a patient-derived xenograft model of GBM, Randall et al. demonstrated varying concentrations of erlotinib that were highest at the tumor core but with limited concentrations at the infiltrative edge and at the interface between tumor and white matter [49]. The variable biodistribution of the drug, specifically low at the tumor edge, remains a clear set up for treatment failure.

## Novel Therapeutics

The BBB also comprises efflux transporters that restrict permeability beyond the physical restrictions imposed by the endothelial cell layer. For example, most of the drugs acting on the PI3K pathway, such as erlotinib, everolimus, gefitinib, and lapatinib, are substrates of two main efflux transporters (P-gp and/or BCRP) expressed at the BBB and demonstrated disappointing results in clinical studies [50–55], at least in part due to inadequate drug penetration into tumor. A retrospective pharmacokinetic analysis of lapatinib after a failed Phase I/II clinical trial revealed that therapeutic concentrations of the drug were not reached [56]. With these failed clinical trial results in mind, current drug development efforts should critically address BBB permeability at an early stage of drug discovery to ensure future success. While the first two classes of therapeutics in this section (“Blood–brain barrier penetrant drugs” and “Nanoparticles”) have undergone more detailed study of BBB penetration, the latter two classes of therapeutics (“Antibody–drug conjugates” and “Radioimmunotherapy”) hold significant promise but may be expected to have inadequate BBB penetration due to their larger size, prompting use of novel drug delivery methods.

### Blood–Brain Barrier Penetrant Drugs

**Fig. 1:**
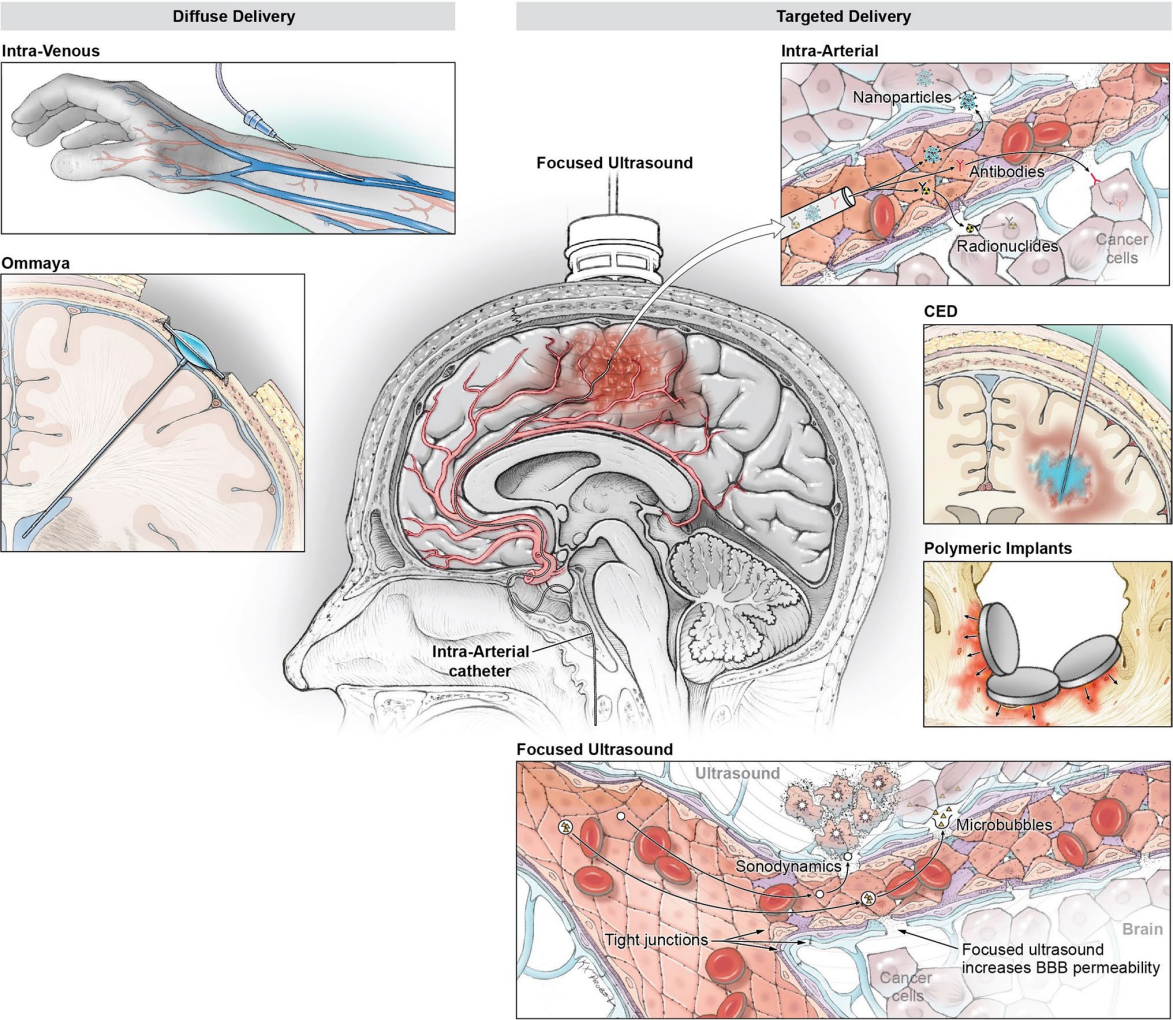
Drug delivery mechanisms for GBM treatment can be broadly categorized into diffuse delivery (e.g., intravenous or via an Ommaya catheter into the CSF) and targeted delivery (e.g., intra-arterial delivery via selective catheterization, convection-enhanced delivery (CED) targeting the tumor, or polymeric wafers implanted in the resection cavity). These methods can be utilized to deliver traditional chemotherapeutic agents as well as novel drugs, such as nanoparticles, antibody–drug conjugates, or radioimmunotherapy. Focused ultrasound can be utilized to disrupt the blood–brain barrier and ultimately increase delivery of these molecules

Chemical modification of drugs to increase BBB permeability can be used to create drugs or prodrugs with enhanced BBB penetration (Fig. 1). Typically, a drug with known antineoplastic effects is conjugated to a chemical moiety that increases its solubility or cell permeability. Release of the active drug is then controlled by unique environmental conditions such as pH, enzyme distribution, and transporter expression.

Paxalisib (GDC-0084) is an example of a small-molecule with specifically designed and optimized physicochemical properties for brain penetration. Paxalisib functions as a dual PI3K and mTOR kinase inhibitor except with high-level penetration across the blood–brain barrier, theoretically rendering greater effectiveness. Surgical samples have confirmed brain tumor-to-plasma and brain tissue-to-plasma ratios of > 1.43 and > 1.54 for total drug and > 0.48 and > 0.51 for free drug, respectively [57]. Although testing in orthotopic models of GBM suggested effectiveness [58], promising results in GBM AGILE were not achieved and paxalisib will not move forward to stage 2 in that trial.

Angiopep-2 is a proprietary 19-amino acid peptide designed to cross the BBB via transcytosis by binding the low-density lipoprotein receptor-related protein (LRP), one of the most highly expressed receptors on the surface of capillary endothelial cells at the BBB, which is upregulated by GBM cells. GRN1005 consists of three paclitaxel molecules covalently linked to Angiopep-2. In a substudy, this drug was detected in the primary brain tumor samples of patients who received GRN1005 4 to 6 h prior to debulking surgery, indicating that GRN1005 successfully crossed the BBB and entered the tumor [59]. While GRN1005 remains a promising drug for the treatment of leptomeningeal carcinomatosis in metastatic Her2-breast cancer, ongoing applications specifically for GBM treatment have halted.

Other chemicals engineered for optimal brain penetration rely on exploiting existing mechanisms of barrier entry, including carrier-mediated transport, receptor-mediated transcytosis, adsorptive-mediated transcytosis, and cell mediated transport. ANG1005, for example, is a promising peptide compound platform technology consisting of three paclitaxel molecules covalently linked to Angiopep-2. ANG1005 was engineered to enter the brain by targeting low-density lipoprotein receptor-related protein (LRP), one of the most highly expressed receptors on the surface of capillary endothelial cells at the BBB, which is upregulated by GBM cells. Results from phase I and II clinical trials are awaited, although early clinical results are promising [60].

### Nanoparticles

Nanoparticles can be delivered past the BBB using various strategies (Fig. 1) and typically can be engineered to help drugs cross the blood–brain barrier without the need to modify the drug itself. Delivery of nanoparticles across the blood–brain barrier is broadly mediated by two methods: passive accumulation of plain nanocarriers or active targeting of the BBB via ligands on their exosurface that imitate biological entities. Polybutylcyanoacrytlate (PBCA) nanoparticles, for example, can be coated with a surfactant pilysorbate 80, which causes absorption of plasma apolipoprotein E, enabling recognition by LDL-receptor expressed in the brain endothelial cells, and ultimately transcytosis. The diverse nature of nanoparticles and their abilities to cross the BBB and to potentially respond to the tumor microenvironment make them a versatile platform for drug delivery.

Limitations to the use of nanoparticles include poor stability of liposomes, poor biocompatibility, low tumor retention, and suboptimal drug release control. Additionally, characterization and validation of complex nanoparticles can be challenging due to the number of parameters to address, such as size, morphology, charge, purity, drug encapsulation efficiency, coating efficiency, and density of conjugated ligands. Despite these challenges, ongoing efforts continue to optimize this approach.

### Antibody–Drug Conjugates

Antibody–drug conjugates (ADCs) are antibodies linked to a payload with inherent anti-neoplastic activity, such as small molecules, protein toxins, biologically active peptides, enzymes, or radionuclides. Although typically administered systemically, the specificity of the targeting antibody enables delivery of the payload to tumor cells selectively. Theoretical advantages include reduced toxicity, enhanced cytotoxicity due to the ability to deliver higher concentrations of drugs that would be toxic if administered systemically, and the synergistic benefit of combined tumor kill from the antibody and the payload. Cell-surface antigens expressed on GBM that have been resistant to signaling inhibition approaches, such as tyrosine-kinase inhibitors or naked monoclonal antibodies, have become attractive targets. Twelve ADCs have been approved by the US Food and Drug Administration (FDA) [61–63], and more than 100 ADCs are currently in clinical trials [64]. For GBM specifically, three ADCs have advanced to clinical trials, namely, depatuxizumab mafodotin (Depatux-M or ABT-414), Abbv-221, and AMG-595. Unfortunately, these studies have been terminated or discontinued. INTELLANCE, a pivotal phase 3 trial evaluating Depatux-M in patients with newly diagnosed GBM, demonstrated no survival benefit [65]. Even though ABBV-221 demonstrated greater treatment efficacy than could be achieved with Depatux-M in a preclinical study, a phase 1 study raised concerns for its safety [66]. Similarly, although AMG 595 was found to be effective in GBM xenograft animal models and had favorable results during a phase I clinical study, the development of AMG-595 was discontinued because of limited efficacy [67, 68]. Limited blood–brain barrier penetration and the immunosuppressive tumor microenvironment may have contributed to these limited results. Ongoing investigative work is focused on resolving these challenges and exploring other ways of implementing ADCs, such as targeting the tumor microenvironment instead of tumor cells. For example, Offenhäuser et al. demonstrated that the EphA3 antibody IIIA4 conjugated with the cytotoxic microtubule-targeting agent maytansine was effective in inhibiting tumor growth in orthotopic xenograft models of glioma [69].

### Radioimmunotherapy

Radioimmunotherapy (RIT) refers to a strategy in which an antibody conjugated to an alpha- or beta-particle emitting radionuclide, referred to as a radioimmunoconjugate (RIC), effects tumor cell death, and/or imaging of biodistribution. RICs can selectively bind to malignant cells leading to cell death from their emitting energy while sparing normal cells. Beta-emitters (e.g., iodine-131 (^131^I), yttrium-90 (^90^Y), lutetium-177 (^177^Lu), rhenium-188 (^188^Re), and iodine-125 (^125^I)) have a longer half-life and therefore have been considered more suitable for intravenous administration and slower uptake into the tumor. Alpha-emitters (e.g., actinium-225 (^225^Ac), bismuth-213 (^213^Bi), and astatine-211 (^211^At)) have a shorter half-life, higher linear energy transfer (LET), and smaller range in tissue and therefore have been preferred for local administration, such as direct application to the surgical bed or intra-arterial delivery.

The commonly employed delivery mechanism is direct administration in the resection cavity as a bolus after tumor debulking [70–72]. This method bypasses a large part of the BBB while minimizing systemic toxicity that may arise from intravenous administration. To overcome the limitations of passive diffusion through dense brain tissue, convection-enhanced delivery (CED), discussed later, has also been employed to enhance local delivery to the tumor site. Other ways of improving drug penetration involve using smaller antibody fragments, such as single-chain variable fragments (scFv) or antibody-derived peptides, since their smaller molecular size enables them to pass through the BBB more effectively.

Despite promising preclinical results for RITs, the small size of clinical trials performed to date has limited interpretation of the results. The largest single study of RIT targeted EGFR with 3 cycles of a total of 140–150 mCi of ^125^I-MAb 425 after debulking surgery and radiotherapy in 192 patients and demonstrated a median survival of 14.5 months in the RIT alone group (*n* = 132) vs. 20.2 months in the RIT combined with TMZ group (*n* = 60) [73]. Other smaller trials have studied RICs targeting tenascin or substance P that yield acceptable safety profiles but limited efficacy [74]. These limited data underscore the importance of combination strategies to overcome radioresistance and intratumoral heterogeneity. Radioimmunotherapy therefore holds greatest promise as a locally delivered therapy in conjunction with multimodal treatment, after careful selection of antigen targets that are highly specific and highly expressed in GBM.

## Locoregional Approaches to Therapeutic Delivery

As limited blood–brain barrier penetration with current drug treatments have come to light, there has been a renewed interest in novel drug delivery mechanisms, such as intra-arterial delivery, convection-enhanced delivery, focused ultrasound, and laser interstitial thermal therapy.

### Intra-arterial Delivery

In recent years, endovascular therapy has emerged as a standard treatment approach for ischemic and hemorrhagic stroke, as well as other malignancies including retinoblastoma and hepatocellular carcinoma [75]. These advancements have renewed interest in utilizing intra-arterial delivery as a potential mechanism for treating GBM. A primary advantage of intra-arterial drug delivery is the ability to administer high doses of therapeutic agents with a limited volume of distribution. This enables targeted and precise delivery of therapeutics to the tumor and surrounding tissues with a shared vascular supply, while minimizing adverse systemic effects.

Another benefit of intra-arterial delivery is the opportunity to prime the brain tumor with chemical agents that locally disrupt the blood-tumor barrier and blood–brain barrier (BTB-BBB), thereby enhancing drug penetration. Traditionally, a hyper-osmolar solution such as mannitol has been utilized to create an osmotic gradient, promoting water movement out of endothelial cells and increasing permeability. In addition to mannitol, other agents such as calcium channel blockers, vasoactive peptides, and nitric oxide donors are also being explored to enhance BBB permeability during intra-arterial drug delivery. These agents target different mechanisms involved in BBB regulation, such as tight junction proteins, transporters, and endothelial cells, and may have varying effects on BBB permeability. Both human and animal trials have shown that chemotherapy concentration can increase 2- to fivefold through intra-arterial delivery when coupled with hyperosmolar therapy [76–78].

Factors that influence arterial concentrations include regional blood flow, injection parameters, the kinetics of BBB transit, and the inherent flow patterns of the tumor’s vasculature. Familiarity with these parameters is important to maximize drug efficacy and minimize the side effect profile. Overall, the rate and severity of complications associated with intra-arterial delivery are not significantly different from those observed during diagnostic cerebral angiograms, with severe long-term complications being uncommon [79, 80]. Ongoing research and optimization of intra-arterial drug delivery parameters, along with the exploration of additional agents, offer potential avenues to improve treatment outcomes in GBM. Phase I clinical trials are ongoing to study intra-arterial administration of temsirolimus (NCT05773326), yttrium-90 microspheres (NCT05303467), TMZ (NCT01180816), cetuximab (NCT01884740, NCT02800486, and NCT02861898), and bevacizumab (NCT01884740, NCT02285959, NCT01811498, and NCT01269853) in recurrent GBM patients.

### Convection-Enhanced Delivery

Convection-enhanced delivery (CED) is another method of delivering drug agents in a more targeted fashion that bypasses the BBB and blood–brain tumor barrier (BBTB). Utilizing Ommaya or Rickham reservoirs or catheters implanted at the time of tumor resection, therapeutic agents can be administered at high concentrations in large volumes over an extended period of time with minimal systemic toxicity [81].

Some of the first studies investigating CED for GBM focused on chemotherapies that are unable to cross the BBB via traditional intravenous administration. One trial explored CED of paclitaxel in 15 patients with recurrent high-grade glioma and reported a median survival of 7.5 months with an imaging response observed in the majority of the treated patients [82]. Another Phase Ib study explored CED of topotecan in recurrent high-grade gliomas and reported radiologic evidence of treatment effect and a median OS of 60 weeks [83]. A 2022 Phase Ib clinical trial in five human GBM patients showed that chronic CED of topotecan through an implantable and programmable subcutaneous pump was a safe therapy for recurrent GBM [84]. Chronic CED is thought to result in improved tumor control by targeting tumor cells that were either not dividing or failed to receive adequate drug during the initial CED. Other strategies have employed CED to deliver conjugated toxins, such as transferrin conjugated to diphtheria toxin (TF-CRM107) or IL-4 conjugated to pseudomonas exotoxin (NBI-3001) [85, 86]. Another large trial compared CED of IL13Ra2 conjugated to a truncated pseudomonas exotoxin with Gliadel wafers for patients with recurrent GBM undergoing resection and found no difference in overall survival [87]. A high-profile trial investigating CED of a live attenuated poliovirus type 1 vaccine with a cognate internal ribosome entry site replaced with a human rhinovirus type 2 protein (PVSRIPO) enrolled 61 patients with recurrent GBM and reported an OS a little over 12 months, with approximately 1 in 5 patients reporting a Grade 3 or higher adverse event related to the treatment [88]. Current CED trials underway include liposomal irinotecan in high-grade gliomas (NCT02022644) and MDNA55 (an interleukin 4 receptor targeting toxin) in recurrent GBM (NCT02858895).

More recent CED studies take advantage of a “step-off” design in the catheter to try to minimize backflow, which is a major limitation with CED therapy. In addition to backflow/reflux, several other challenges have been identified in the clinical setting. For example, this delivery mechanism requires a priori planning, as it requires surgical implantation ideally at the time of the resection. Second, there is a learning curve for optimal catheter placement. In one of the major trials (PRECISE), less than 70% of cannulas were positioned in accordance with protocol guidelines, as catheter placement should typically be 2 cm away from the pial surface or resection cavity to minimize the risk of backflow. Third, monitoring of the infusate distribution in the tumor was somewhat limited. Finally, when the tumor is located near the ventricular lining or has a large cystic cavity, the infusate will distribute into these lower pressure gradient areas and leave the areas of more viable tumor, which typically have higher interstitial pressures, uncovered by infusate [81].

### Laser Interstitial Thermal Therapy (LITT)

LITT utilizes an optical fiber, which is placed with stereotactic or image guidance in the tumor through a burr hole. The primary goal is to ablate tumor cells via thermal energy. Heating typically occurs utilizing a wavelength of 1064 nm or 980 nm to heat to 42.5–45.5 °C for several minutes. This technique is particularly attractive for patients with deep-seated tumors or patients with medical comorbidities that make more invasive resections risky. Studies evaluating LITT in GBM have been primarily limited to patients with tumors < 50 mm in diameter with residual or recurrent disease [89]. Extent of ablation appears to be an important determinant of its efficacy with near-total ablation correlated with improved PFS and OS [90, 91]. Retrospective reports have reported variable efficacy and complication rates can be as high as 33% in newly diagnosed GBM, which is higher than those typically reported with open cytoreduction approaches [92]. LITT may have limited efficacy for GBM located near the ventricles (due to the heat sink effect), large GBM in proximity to the brainstem (where relief of mass effect may be needed), and GBM near the pial surface (where open resection may be advantageous). The recently published LAANTERN prospective trial (NCT02392078) investigated LITT in patients with newly diagnosed or recurrent GBM and reported median ablations between 91 and 99% with median OS 9.73 months for newly diagnosed patients [93]. In this trial, the adverse event rate was 13.5% with neurological deficits being the most common adverse event reported. Tumor volumes < 3 cc were associated with improved survival. The average length of hospital stay is shorter following LITT than open resection.

Beyond cytoreduction, studies suggest that LITT can also enhance BBB permeability. Disruption of the BBB is noted to occur 1–2 weeks after LITT with resolution by 4–6 weeks [94], potentially providing a therapeutic window during which additional therapies can be delivered. Additionally, there is evidence that localized hyperthermia via LITT can impact tumor cells directly, modulate immune cell function and activation, and change the tumor microenvironment via the release of tumor antigen-dense exosomes and immune-stimulating heat-shock proteins (HSPs), increased cytokine and chemokine production, and enhanced antigen-presenting cell (APC), cytotoxic T cell, and natural killer (NK) cell activity [95]. These effects can in theory be therapeutically co-opted to convert the tumor environment from an immunosuppressed “cold” state to a “hot” state that is more responsive to checkpoint blockade or adoptive T cell therapies.

### Focused Ultrasound

Focused ultrasound (FUS) is an emerging, non-ionizing technique in which ultrasound waves are delivered and focused on a target to produce a variety of biological effects enabling noninvasive treatment. Until recently, FUS applications for the brain were hindered due to the strong attenuation and distortion of ultrasound waves by bone from an extreme impedance mismatch and limited application through an intact skull. This can be circumvented through use of ultrasound devices implanted in place of the craniotomy bone flap. However, several technological developments have also enabled noninvasive application of FUS through an intact skull: (1) use of lower ultrasound frequencies in the sub-megahertz range that better penetrate the skull, (2) development of low-frequency, large, multi-element phased arrays that allow for electronic correction of beam distortion by individual adjustment of the phase of each acoustic wave, (3) calculation of phase and amplitude corrections needed from patient-specific skull CT data, and (4) intra-procedural MRI-based imaging and thermometry guidance [96–98].

Currently, FUS has four main treatment applications discussed below, namely, (1) thermal ablation, (2) histotripsy, (3) blood–brain barrier disruption, and (4) sonodynamic therapy.

#### Thermal Ablation

When FUS is used in a continuous wave mode, it results in the accumulation of thermal energy within the targeted tissue. Temperature of > 56° for 2 s or more can result in irreversible cell death through coagulative necrosis [99, 100]. This property can be exploited in ablative treatments for symptomatic uterine fibroids [101, 102]; tumors in the prostate, breast, and liver [103–105]; low back pain [106]; and brain disorders such as essential tremor, Parkinson’s disease, and neuropathic pain [107–109].

In ablative treatment for essential tremor, the dentato-rubro-thalamic tract at the ventral intermediate thalamus is targeted. A randomized, controlled trial involving 76 patients with medication-refractory essential tremor showed that transcranial FUS thalamotomy significantly reduced hand tremor at 3 months, and the effect persisted during the 12-month study period [108]. Since this publication in 2016, other academic centers have seen similar success rates for the treatment of tremors via this method.

The application of ablative ultrasound technology for brain tumors remains a challenge. In 2010, McDannold et al. managed, for the first time, to focus the ultrasound beams transcranially into the brain and visualize heating with an MR temperature imaging system in three GBM patients. However, they were unable to reach a complete tumor ablation due to the low power of the FUS device, and the trial was stopped when a fourth patient suffered a cavitation-induced fatal intracranial hemorrhage [110]. Five different studies investigating the effects of FUS in direct tumor ablation for treating HGG patients (*n* = 23) have shown similar difficulties and high complication rates, with more than one patient out of four developing a hematoma at or near the FUS target [111]. Most available data is from case series reports or preliminary reports, and at the moment, there is insufficient data on patients’ neurological outcomes.

FUS ablative treatment of pediatric brain tumors has shown more promise. Published results from the first five patients in a clinical trial using FUS to treat benign pediatric brain tumors, namely, hypothalamic hamartomas and a subependymal giant cell astrocytoma, show that ablation of these tumors was feasible without major medical, neurological, or endocrinological adverse events for on average 25 months and without radiologic complications for up to 12 months [112].

#### Histotripsy

When ultrasound waves are delivered at high acoustic intensities with a short pulse duration, as opposed to the continuous wave mode, non-thermal effects predominate. Microbubbles will form around cells and disrupt the cell membrane through their oscillations—a process known as inertial cavitation. Inertial cavitation can also release shockwaves that ultimately liquefies cells, in a process known as histotripsy. A unique advantage of histotripsy over other modalities of FUS is that the microbubbles are easily imaged, allowing for easy localization of the focal region of the FUS beam during MRI guidance [113]. This technique has been used to liquefy large clots in an intact skull [114]. Other clinical applications include its use to treat liver tumors and to soften plaque in calcified aortic stenosis [115]. Even though histotripsy has high potential to treat GBM, it has not been extensively studied for this purpose, with very limited in vivo trials and no current clinical trials at the moment. The high intensity requirements of histotripsy mean that a craniotomy would be required before sonication, limiting the exploration of this technique as a viable treatment option currently.

#### Blood–Brain Barrier Disruption

When ultrasound is delivered in short pulse duration at intensities lower than the threshold for tissue destruction, stable cavitation as opposed to inertial cavitation occurs near the targeted cells. The less violent oscillations of these microbubbles can temporarily open pores in the cell membrane, in a process known as sonoporation, as well as reduce the integrity of inter-endothelial tight junctions, and increase transcellular vesicular trafficking. This transient permeability can be used as a means to enhance drug delivery in targeted regions of the brain. Additionally, there is data suggesting that BBBD with FUS transiently disrupts P-glycoprotein (P-gp), the most dominant multi-drug-resistant efflux transporter found in the BBB, which normally acts to pump out many xenobiotic molecules into the blood [116].

In an orthotopic murine glioma model, the concentration of etoposide in tumor tissue was found to increase by almost eightfold after FUS-mediated BBB disruption. More importantly, treatment with this technique led to a 45% decrease in tumor growth as well as a 30% improvement in median survival [117]. In humans, a Phase 0 clinical trial involving four patients demonstrated that noninvasive BBB opening can be performed safely [118]. Another small clinical trial, comprising six patients with GBM who underwent multiple cycles of BBB disruption using MRgFUS to enhance the penetration of TMZ chemotherapy, also found that MRgFUS is safe and well-tolerated without any significant long-term complications at 1-year follow-up [119].

The promising results obtained from preclinical and clinical studies suggest that FUS-induced BBB disruption has the potential to revolutionize the treatment of GBM. Ongoing trials for FUS are evaluating whether BBB disruption with FUS can improve the effects of systemic chemotherapy (NCT03712293 and NCT03551249) or radiotherapy (NCT04988750).

#### Sonodynamic Therapy

Low-intensity ultrasound can also be used to activate tissue that has been sensitized with a non-toxic chemical agent. While both sensitization and ultrasound exposure are harmless individually, the combination of both results in cytotoxic events which can be exploited for targeted treatment. One of the popular sensitizers that has been studied is 5-aminolevulinic acid (5-ALA), a porphyrin-based compound. When 5-ALA is administered exogenously, one of its deriving porphyrins—protoporphyrin IX (PpIX)—preferentially accumulates in the intracellular compartment of tumor cells due to decreased levels of ferrochelatase and selective uptake by an ATP-binding cassette transporter (ABCB6) [120]. Low-intensity focused ultrasound can generate sonoluminescence that activates PpIX and results in the formation of cytotoxic reactive oxygen species. Focused ultrasound delivered in rat 9L glioma models treated with 5-ALA was found to significantly reduce tumor size while not effecting the histological integrity of the surrounding normal brain [121]. Similar results were also seen in in vivo porcine models of intracranial gliomas [122].

The selective accumulation of a 5-ALA derivative in tumor cells is already exploited in surgical planning, where fluorescence-guided surgery (FGS) permits intraoperative visualization of malignant glioma tissue to help differentiate tumor from normal brain in real time [123]. As a result, there has been tremendous interest in the clinical translation of sonodynamic therapy for these patients. Preliminary results from the first-in-human clinical trial of MR-guided FUS SDT with 5-ALA in glioma patients show that treatment is safe at 200 J and that sonodynamic therapy leads to targeted oxidative stress and tumor cell death in human GBM tissue, as evidenced by elevated biomarkers of oxidative stress (4-hydroxynonenal, glutathione, cysteine, and thiol) and apoptosis (cleaved caspase-3) in treated tissue versus internal controls [124].

## Clinical Trials and Future Directions

Clinical trials of novel GBM therapies are frequently marred by shortcomings in design that jeopardize understanding of a drug’s optimal dosage, safety, and effectiveness, many of which relate to aforementioned challenges with unpredictable penetration of drugs through the areas of intact BBB in GBM. Addressing deficiencies in trial design will be crucial to ensure optimal resource utilization and efficient drug discovery. For example, the premature promotion of the PKC and PI3K/AKT inhibitor enzastaurin to phase III was based on early imaging results showing a relatively high objective radiologic response rate of 20%. This led to additional trials which proved to be unsuccessful [125]. Numerous trials and reviews have shared similar cautionary tales of failed late-stage trials, underscoring the importance of well-designed early-phase studies to address critical questions [126, 127].

In light of these deficiencies, we advocate for (1) neoadjuvant and window-of-opportunity studies (WoO) to confirm drug delivery and pharmacodynamic impact of the therapy on tumor biology; (2) intelligent imaging endpoints that integrate drug delivery, metabolic, mechanistic, and response criteria; (3) adaptive platform trials; and (4) basket trials as helpful adjuncts to traditional clinical trial design. These trial designs, while not new, have not been fully embraced by all investigators and sponsors. These are important to recognize as suitable approaches for GBM where treatment paradigms are growing increasingly complex. For example, a trial may need to assess the best delivery methodology for a novel agent, delivered intra-arterially, intravenously, or intrathecally with or without FUS or LITT BBB disruption. For these more complex treatment paradigms, these alternative trial designs are more appropriate.

### Neoadjuvant and Window of Opportunity Trials

Neoadjuvant and window-of-opportunity (WoO) studies consist of several key steps: (1) confirmation of the tumor diagnosis via biopsy specimen, (2) treatment with a therapeutic agent, (3) tissue retrieval via surgery, and (4) assessment of tumoral response.

**Fig. 2:**
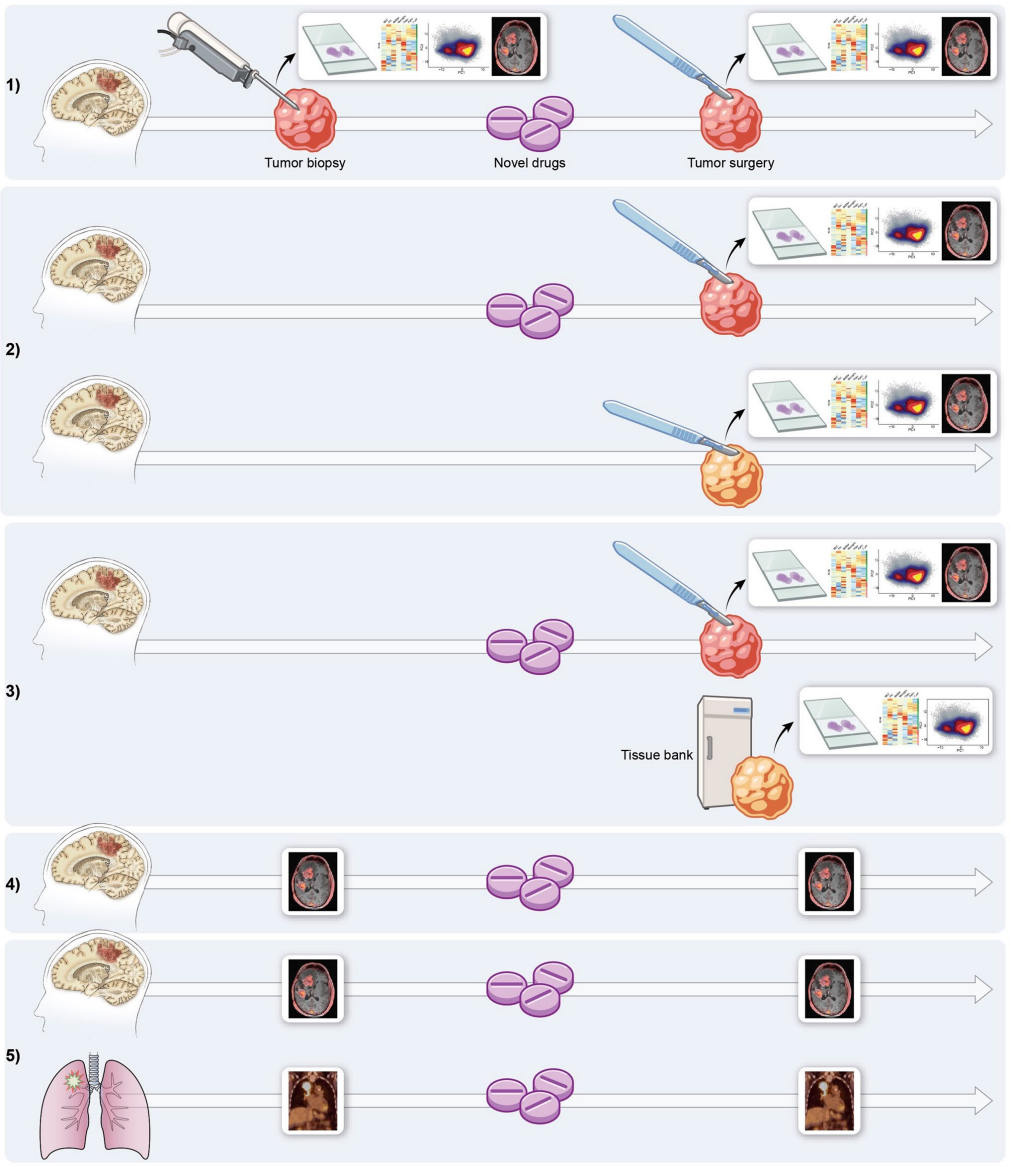
Clinical trial designs. *Window of opportunity trial designs*: (1) all patients undergo pretreatment biopsy, pre-treatment imaging, a novel treatment, post-treatment surgery, and post-treatment imaging. Surgical specimens are assessed and compared using standard assays. Pre- and post-treatment imaging is compared. (2) Patients do not undergo biopsy but are randomly assigned to one of two groups. One group receives the treatment before surgery, while the other does not. Both groups then undergo post-treatment resection of the tumor and post-treatment imaging. Tumor specimens and imaging from untreated and treated patients are assessed and compared. (3) All patients receive imaging and treatment and undergo surgery; tissue-bank specimens serve as a control. (4) All patients receive pre-treatment imaging, treatment, and post-treatment imaging. Pre- and post-treatment imaging is compared. *Basket trial design*: (5) tumors with similar genetic makeups are grouped together even if they come from different organs. All patients undergo a treatment. Biopsy and surgery specimens are assessed and compared using standard assays. Pre- and post-treatment imaging is compared

In a neoadjuvant trial, the novel therapeutic is typically administered for a longer period of time prior to surgery, and the goal is to document a measurable pathologic or clinical response [128]. By contrast, in WoO trials, the novel therapeutic is typically administered for a short period of time so as to not delay standard-of-care treatment; the goal is to utilize the acquired after-treatment tissue to determine the presence of the tested therapeutic its effect on the tumor and its microenvironment (Fig. 2). The tissue also provides researchers with the potential to identify key biomarkers that can serve as endpoints to assess treatment response more faithfully [129]. In contrast to phase 0 trials, WoO trials occur after phase I evaluations and typically use higher drug concentrations than the sub-therapeutic microdoses used in phase 0 trials to establish human pharmacokinetics and the suitability of drug candidates to advance to phase 1.

### Adaptive Platform Trials

Adaptive platform trials help evaluate multiple therapies simultaneously with the goal of efficiently and rapidly identifying effective therapies. If one treatment arm outperforms another, a higher proportion of new enrollees is assigned to that treatment arm—a technique termed “adaptive randomization” [130]. Drugs that show initial evidence of benefit to patients will transition to a confirmatory stage designed to support drug approval, and drugs that underperform are dropped. As such, this type of adaptive platform trial allows researchers to utilize data connectivity within the trial to answer many questions concurrently, while testing different drug paradigms, without wasting tremendous resources [130]. A similar clinical trial design could be utilized to test one drug across multiple drug delivery platforms. The GBM AGILE trial (NCT03970447) is an ongoing platform trial that assesses drug agents specifically for GBM. The first drug to be evaluated was regorafenib. The regorafenib investigational arm was concurrently and adaptively randomized against other investigational arms, and regorafenib was ultimately dropped after interim analysis showed a low probability of sufficient improvement in OS as compared with randomized controls. The design of GBM AGILE allows for efficient allocation of resources to alternative treatments that may be more efficacious. A similar clinical trial design could be utilized to test one drug across multiple drug delivery platforms.

### Basket Trials

Current drug development is dominated by efforts to create therapies that target specific molecular aberrations, which can be shared across a variety of tumors originating from different organs. Basket trials are tissue-agnostic trials assessing drugs that target a common pan-cancer gene defect. There are several prototypes of basket trials, the most common being the following three: (1) disease-specific baskets (e.g., dabrafenib was targeted against BRAF in a variety of cancers with different primary disease sites [131]); (2) disease-mutation-specific baskets (e.g., CREATE trial, crizotinib inhibits multiple oncokinases including c-Met and anaplastic lymphoma kinase, and the baskets reflect a combination of diseases and targets); and (3) disease-drug-mutation-specific baskets (e.g., CUSTOM trial, five targeted therapies are tested on patients with one of three diseases, resulting in 15 disease-drug-mutation–specific baskets) [132]. The benefit of basket trials is that they are an efficient way of assessing outcomes in diseases that may otherwise be underpowered, either due to their rarity or due to the low number of patients enrolled in the trial. However, careful study design and implementation are important. A basket study that implements multiple independent two-stage designs will have a much higher false-positive rate than a typical phase II study and therefore a higher likelihood of being declared effective in at least one basket when in fact the drug is truly ineffective [132].

### Imaging and Clinical Trials

Classically, standard phase I and II clinical trials determine the safety and efficacy of agents by using indirect global end points. Optimal therapeutic dose of an agent is identified by acceptable levels of systemic or neurologic toxicity as end points, and efficacy is identified radiologically by monitoring enhancing tumor, a surrogate for tumor volume. Unfortunately, this ignores imaging data regarding biodistribution of the administered agents, target engagement by the agent, immune system activation, and other critical imaging biomarkers that may precede or predict an objective radiologic response in terms of tumor size. We propose the early integration of advanced imaging techniques, particularly those informed by the trial therapy’s mechanism of action, instead of or in addition to the traditional contrast-based Response Assessment for Neuro-Oncology (RANO) criteria, as imaging endpoints. While not novel ideas, integration of these more advanced biomarkers within drug trials has lagged.

Integration of imaging in clinical trial design has historically been done by way of using morphologic and anatomic imaging to assess a tumor’s response to a drug agent, usually at standard of care imaging time points using RANO criteria [133]. The molecular era and rise in targeted therapy for gliomas has led to increased utilization of advanced imaging techniques; however, these techniques are typically applied to resolve ambiguities associated with treatment response (i.e., pseudoprogression versus tumor progression) and are not integrated as outcome metrics in clinical trial assessment. Integration of molecular imaging techniques, such as positron emission tomography (PET) that assesses the biologic process targeted by the investigational therapy, has largely been exploratory. These exploratory aims are not powered to generate meaningful conclusions and can at best be used to show associations, although, ironically, these imaging techniques carry the most potential for deriving biologic insight into modes of therapy failure or resistance. As a result, there is a great need to integrate imaging specialists early in trial design, in order to identify appropriate imaging metrics that are pragmatic but also scientifically informed. While trials have focused primarily on tissue acquisition or imaging assessment utilizing RANO criteria, we propose early implementation of advanced imaging techniques, such as hybrid PET/MRI, perfusion MRI, MR spectroscopy, and artificial learning algorithms, as imaging methods that could help better elucidate treatment response. Early implementation of advanced imaging could be used within any of the proposed trial designs and may in particular be useful in combination with WoO trial design (Fig. 2).

At the moment, however, there are real and important challenges for integration of advanced imaging techniques into clinical trial assessment, which include considerations for siting of imaging equipment, specific hardware and software requirements, being able to provide maintenance of the hardware and software, reliance on staff with the appropriate niche technical expertise, logistics on how to process and store the acquired data, and importantly, the high financial costs incurred by these components. With appropriate foresight and resources, however, these hurdles can be overcome and will better inform the results of future clinical trials.

## Conclusion

In this review, we have discussed the biological traits of GBM and the BBB that pose challenges for the development of current therapeutic agents with an emphasis on novel drugs and alternative methods of drug delivery, and the ongoing challenges of drug design, positing combination therapy and novel trial designs with early integration of on-treatment tumor tissue analysis and imaging biomarkers as optimal strategies for early trial designs moving forward. This review hopes to provide context and guidance for those seeking an informed approach to development of the next generation of clinical trials in treatment of this challenging disease.

## Data Availability

No datasets were generated or analyzed during the current study.
